# Serum d-serine levels are altered in early phases of Alzheimer’s disease: towards a precocious biomarker

**DOI:** 10.1038/s41398-021-01202-3

**Published:** 2021-01-26

**Authors:** Luciano Piubelli, Loredano Pollegioni, Valentina Rabattoni, Marco Mauri, Lucia Princiotta Cariddi, Maurizio Versino, Silvia Sacchi

**Affiliations:** 1 Department of Biotechnology and Life Sciences, University of Insubria, Varese, Italy; 2 Neurology Unit, Ospedale di Circolo and Fondazione Macchi, ASST Settelaghi, Varese, Italy; 3 Center of Research in Medical Pharmacology, University of Insubria, Varese, Italy; 4 Department of Medicine and Surgery, University of Insubria, Varese, Italy

**Keywords:** Diagnostic markers, Molecular neuroscience

## Abstract

d-Serine acts as a co-agonist of *N*-methyl-d-aspartate receptors (NMDAR) which appear overactivated in AD, while d-aspartate is a modulatory molecule acting on NMDAR as a second agonist. The aim of this work is to clarify whether the levels of these d-amino acids in serum are deregulated in AD, with the final goal to identify novel and precocious biomarkers in AD. Serum levels of l- and d-enantiomers of serine and aspartate were determined by HPLC using a pre-column derivatization procedure and a selective enzymatic degradation. Experimental data obtained from age-matched healthy subjects (HS) and AD patients were statistically evaluated by considering age, gender, and disease progression, and compared. Minor changes were apparent in the serum l- and d-aspartate levels in AD patients compared to HS. A positive correlation for the d-serine level and age was apparent in the AD cohort. Notably, the serum d-serine level and the d-/total serine ratio significantly increased with the progression of the disease. Gender seems to have a minor effect on the levels of all analytes tested. This work proposes that the serum d-serine level and d-/total serine ratio values as novel and valuable biomarkers for the progression of AD: the latter parameter allows to discriminate CDR 2 and CDR 1 patients from healthy (CDR 0) individuals.

## Background

Alzheimer’s disease (AD), the most common cause of late-onset dementia, is a chronic and progressive neurodegenerative disease affecting ≈6% of adults over 65 years of age^1^. The preliminary diagnosis of AD is made by a combination of clinical criteria, which includes neurological examinations, mental status tests, and brain imaging. On the basis of these clinical tests only, however, diagnosis of AD becomes a difficult task, especially in the early pre-symptomatic phase of the disease. Currently, analysis of the cerebrospinal fluid (CSF) with established biomarkers^2,3^ is carried out for research purposes. However, this fluid is obtained by an invasive and painful procedure: there is an extreme need for alternative, easily detectable biomarkers, which may also be sensitive and specific^4^.

AD pathophysiology is characterized by the accumulation of extracellular amyloid β (Aβ) plaques and intraneuronal inclusions of the truncated and phosphorylated forms of tau protein (neurofibrillary tangles). This appears to induce dystrophic neurites, loss of synapses, a prominent gliosis (involving changes in the morphology and function of microglia and astrocytes) and, only at later stages, overt loss of neurons and associated brain atrophy^5^. Consistent with the role of the glutamatergic system in learning and memory formation, alterations in *N*-methyl-d-aspartate receptor (NMDAR)-mediated neurotransmission have been linked with the pathological processes underlying AD^6,7^. Accordingly, Aβ induces synaptic dysfunction by perturbing synaptic Ca^2+^ handling in response to overactivation of postsynaptic NMDARs^8^, leading to oxidative stress, spine loss, and gradual neuronal cell death, which in turn correlates with the progressive decline in memory and cognition in AD^7,9^ but also by altering presynaptic functions^10^.

Among the major factors affecting NMDAR-mediated neurotransmission in AD, availability of the agonist glutamate and the modulation of the receptor’s functions are extremely important:^11,12^ the non-competitive NMDAR antagonist memantine has been approved for treatment of moderate to advanced AD^13^. Modulatory molecules playing a role in NMDAR function may also be related to AD. In particular, d-aspartate (d-Asp) acts as a second agonist^14,15^ and d-serine (d-Ser) acts as the main endogenous co-agonist^16^.

In mammals, d-Asp is abundant in the embryonic brain, while during adulthood its levels are extremely low and strictly controlled by the catabolizing enzyme d-aspartate oxidase (DASPO)^17^. The long-lasting exposure to nonphysiological, high concentration of d-Asp in DASPO knock-out mice (*Ddo*^−/−^) elicited a precocious decay of synaptic plasticity and cognitive functions^18^. Moreover, severe processes related to neuroinflammation were observed in this animal model, as indicated by the appearance of dystrophic microglia and reactive astrocytes^19^, distinctive features in neurodegenerative disorders^20,21^. Recently, the high d-Asp levels in *Ddo*^−/−^ mice were also shown to induce changes in tau phosphorylation^22^. Notably, the d-Asp content in tissues and/or biological fluids appeared to be altered in AD patients, despite differing levels having been reported: halved d-Asp levels were detected in the white matter of AD brains compared to healthy subjects (HS)^23^ whereas higher levels were measured in the CSF of AD patients with respect to HS^24,25^. It is remarkable that all these studies were carried out on a limited number of subjects (≤10 for both HS and AD patients).

On the other hand, brain d-Ser is synthesized starting from the corresponding l-enantiomer by the enzyme serine racemase (SR), and is degraded by both SR and d-amino acid oxidase (DAAO, mainly located in astrocytes)^26^. Notably, a strong upregulation of SR was reported in reactive astrocytes in the hippocampus and entorhinal cortex of subjects with AD, which increased with disease progression: large part of these astrocytes were also neurotoxic^27^. Aβ aggregates induced release of d-Ser and its brain content was increased in the animal models of the disease^28–30^, suggesting that the high d-Ser levels yield excitotoxicity, thus triggering neuronal death in AD. The DAAO inhibitor sodium benzoate improved cognitive and overall function in AD patients with early-phase disease when used at high concentrations for 24 weeks^31^.

Concerning the detection of d-Ser levels in AD patients, during the years contrasting results have been reported. By using traditional high-performance liquid chromatography (HPLC) procedures, Nagata et al.^32^ reported no changes in d- and l-Ser levels in the frontal cortex compared to HS while a decrease in the d-Ser levels, coupled to a slight increase in l-Ser resulting in a significant decrease in d-/total-Ser ratio, was observed in human serum^33^, albeit no details about the experimental procedure used are provided. Differently, an increase in d-Ser and SR levels was reported in post-mortem hippocampal and parietal cortex of AD patients compared to HS^30^. The same work reported higher d-Ser level in the CSF of probable AD than in non-cognitively impaired subject groups by using classical HPLC procedures. These results confirmed previous observations from D’Aniello’s group^25^ performed using an HPLC method coupled to enzymatic degradation. Most recently, an analysis based on ultra-HPLC-tandem mass spectrometry reported tiny changes in the d-Ser and d-/total-Ser levels^34^.

In our opinion, these confounding results are largely ascribable to the absence of standardized protocols and suitable controls. For this reason, this investigation is aimed at clarify whether d-Ser and d-Asp levels in serum are deregulated in AD using a well-established analytical procedure validated by the use of selective enzyme degradation and following the guidelines reported in ref. ^35^. This with the final goal to propose d-Ser and/or d-Asp as novel and precocious biomarkers in AD useful for detecting the progression of the disease.

## Materials and methods

### Subject recruitment and sample collection

Peripheral venous blood samples were collected from patients with AD and from age- and sex-matched HS recruited from outpatients attending the Alzheimer’s Assessment Unit (CDCD) at the Ospedale di Circolo and Fondazione Macchi in Varese (ASST Settelaghi), Italy. AD was diagnosed according to the NIA-AD criteria^3^ and the disease stage of AD patients was assessed by using the Clinical Dementia Rating (CDR) Scale that allows to characterize five domains of cognitive and functional performance in AD dementia. Scores are defined as follows: 0: HS; 0.5: questionable or very mild dementia; 1: mild dementia; 2: moderate dementia; 3: severe dementia^36^. For this study, only AD patients presenting CDR 1 or 2 were enrolled. HS were caregivers of the recruited AD patients with Mini-Mental State Examination Score ≥ 27/30 not suffering of present or past neurological and/or psychiatric pathologies influencing cognitive functions. The Ethics Committee of the Ospedale di Circolo and Fondazione Macchi of Varese approved the protocol and all participants signed a written informed consent before enrollment. Withdrawal of venous blood was performed after a fasting night, between 8:00 and 10:00 a.m., in BD Vacutainer™ SST™ II Advances Tubes (Becton Dickinson, Franklin Lakes, NJ, USA) including clot activator and gel for serum separation. Tubes were coded and serum separation was performed by centrifugation. Sera were subsequently frozen and stored at −80 °C until HPLC analyses. The AD patients recruited for this study were not affected by serious behavioral disorders; therefore, they were not subjected to the administration of antipsychotic drugs.

### High-performance liquid chromatography analyses

HPLC grade methanol, acetonitrile and tetrahydrofuran were from Honeywell International (Seelze, Germany). All other chemicals and reagents were from Merck Life Sciences (Darmstadt, Germany).

Serum samples were added of HPLC grade methanol (90% v/v final concentration) and vigorously vortexed for 3 min at room temperature to precipitate serum proteins, that were then removed by centrifugation (16,000 × *g* for 15 min at 4 °C). Supernatants were dried, suspended in 0.2 M trichloroacetic acid, neutralized with NaOH, and subjected to pre-column derivatization with *o*-phthalaldehyde and *N*-acetyl-l-cysteine. Separation of the amino acid enantiomers was carried out by reversed-phase HPLC on a Symmetry C8 column (4.6 × 250 mm, bed volume 4 mL) (Waters S.p.A., Sesto San Giovanni, MI, Italy) using a HPLC PU-2089 System (Jasco Europe, Cremella, LC, Italy) equipped with a fluorescence detector, as described in ref. ^37^. Separation was carried out at 1 mL/min, under isocratic conditions in 0.1 M sodium acetate in the presence of 1% (v/v final concentration) tetrahydrofuran at pH 6.2 as the mobile phase. All investigated amino acids were detected in a single run lasting 30 min. A washing step with 10 mL of 0.05 M sodium acetate buffer, 47% acetonitrile and 3% tetrahydrofuran (both v/v final concentrations), pH 6.2, was performed after each run. Identification of d- and l-amino acids was based on retention times obtained with external standards (retention times of 4.3 ± 0.2, 5.1 ± 0.2, 21.2 ± 0.6, and 23.3 ± 0.7 min for d-Asp, l-Asp, d-Ser, and l-Ser, respectively). Peaks identity was confirmed by the selective degradation of the d-enantiomers by RgDAAO M213R variant^38^: the samples were added with 10 μg of enzyme, incubated at 30 °C for 4 h, and then subjected to derivatization and HPLC analyses. Quantification of enantiomers was based on peak areas by means of calibration curves for each enantiomer.

Distribution of the values of variables was assessed by the D’Agostino and Pearson normality test. A non-normal (or suspected non-normal) distribution for d-/l-Asp and d-/l-Ser was observed, both as absolute levels and as ratios: therefore, a non-parametric approach was used for all statistical analyses. Statistical significance of two-samples comparisons of continuous variables were performed using the two-tailed Mann–Whitney test. Correlations among continuous variables were assessed by Spearman non-parametric correlation analyses. The values reported represent mean ± standard deviation, unless otherwise stated. All analyses were performed using GraphPad Prism 7.0 (GraphPad Software, San Diego, CA, USA). A *P* value <0.05 was considered as statistically significant.

## Results

A total of 26 HS from 64 to 86 years and 42 AD patients from 64 to 87 years were enrolled for this study. The demographic and clinical characteristics of the subjects are reported in Table 1. The two groups are age-matched: the mean age was 76.7 ± 5.9 and 79.1 ± 5.4 years (mean ± SD) for HS and AD patients, respectively: statistical analyses showed no significant differences (*P* = 0.0834, Mann–Whitney test). Percentages of females were 53.8% and 66.7% in HS and AD patients, respectively. Mean age values of male cohorts were 76.8 ± 7.2 and 78.5 ± 5.4 years (HS and AD patients, respectively), and those of female cohorts were 76.6 ± 4.8 and 79.4 ± 5.4 years (HS and AD patients, respectively): in these cases also no statistically significant differences have been found comparing mean age values of HS and AD patients (*P* = 0.4687 and *P* = 0.0711 for male and female cohorts, respectively, Mann–Whitney test).

**Table 1:** Demographic and clinical data of the subjects enrolled for the study.

Demographic data	Healthy subjects	AD patients	*P* value
Number	26	42	–
Male (% of total)	12 (46.2)	14 (33.3)	0.2904^*^
Female (% of total)	14 (53.8)	28 (66.7)	
Age, range (years)	64–86	64–87	–
Male, range (years)	64–86	64–87	–
Female, range (years)	66–84	68–86	–
Age, mean ± SD (SEM)	76.7 ± 5.9 (1.16)	79.1 ± 5.4 (0.83)	0.0834
Male, age, mean ± SD (SEM)	76.8 ± 7.2 (2.08)	78.5 ± 5.4 (1.44)	0.4687
Female, age, mean ± SD (SEM)	76.6 ± 4.8 (1.28)	79.4 ± 5.4 (1.03)	0.0711
CDR, mean ± SD (SEM)	NA	1.41 ± 0.50 (0.077)	–
Male, CDR, mean ± SD (SEM)	NA	1.43 ± 0.51 (0.14)	–
Female, CDR, mean ± SD (SEM)	NA	1.39 ± 0.50 (0.094)	–
CDR, number (in parentheses) and age, mean ± SD (SEM)			
CDR 1 (25)	NA	77.9 ± 5.7 (1.15)	0.1012**
CDR 2 (17)	NA	80.9 ± 4.4 (1.06)	
No. of subjects using anti-dementia drugs (% of total)^a^	NA	38 (90.5)	
Memantine, number (% of total)	NA	19 (42.9)	–
Memantine, mg/die, mean ± SD (SEM)	NA	18.4 ± 3.8 (0.86)	–
Donepezil, number (% of total)	NA	14 (33.3)	–
Donepezil, mg/die, mean ± SD (SEM)	NA	7.5 ± 2.6 (0.69)	–
Rivastigmine, number (% of total)	NA	7 (16.7)	–
Rivastigmine, mg/die, mean ± SD (SEM)	NA	6.6 ± 2.1 (0.79)	–

*SD* standard deviation, *SEM* standard error of the mean; *P* values refer to comparison between HS and AD patients (Mann–Whitney test). *NA* not applicable.

*The comparison was performed using the *χ*^2^ test.

**P value refers to the comparison between CDR 1 and CDR 2 AD patients (Mann–Whitney test). *P* values obtained from comparison between HS and AD patients disaggregated by CDR are reported in the text.

^a^Two AD patients were assuming both memantine and donepezil.

The levels of d- and l-aspartate and d- and l-serine in serum were assayed by the pre-column derivatization procedure, confirming the identity and amount of the d-enantiomers (present at a significantly lower level) by a selective enzymatic degradation. Age-related variation of the investigated amino acids was analyzed by Spearman non-parametric correlation analyses (Supplementary Fig. 1): the d- and l-Asp levels and d-/total-Asp ratio did not significantly vary with age, neither in samples from HS nor from AD patients (d-Asp HS: *r* = 0.216, *P* = 0.2891; d-Asp AD: *r* = 0.02573, *P* = 0.8715; l-Asp HS: *r* = 0.03024, *P* = 0.8834; l-Asp AD: *r* = −0.2589, *P* = 0.8707; d-/total-Asp ratio HS: *r* = 0.1133, *P* = 0.5814; d-/total-Asp ratio AD: *r* = 0.02715, *P* = 0.8645). The same analyses showed no correlations with age for d- and l-Ser levels and for d-/total-Ser ratio in samples from HS (d-Ser: *r* = 0.1002, *P* = 0.6264; l-Ser: *r* = −0.2784, *P* = 0.1684; d-/total-Ser ratio: *r* = 0.3844, *P* = 0.525); while a positive correlation has been found both for d-Ser (*r* = 0.5242, *P* = 0.0004) and for d-/total-Ser ratio (*r* = 0.3663, *P* = 0.0170), but not for l-Ser (*r* = 0.2164, *P* = 0.1687), in samples from AD patients.

In order to exclude any effect due to the administration of memantine on the levels of the analyzed molecules, the values obtained for patients assuming and not assuming the drug were compared. In all cases, no significant difference has been observed (d-Ser: *P* = 0.6753; l-Ser: *P* = 0.4872; d-/total-Ser ratio: *P* = 0.7977; d-Asp: *P* = 0.6570; l-Asp: *P* = 0.0918; d-/total-Asp ratio: *P* = 0.3126; Mann–Whitney test). Thus, all patients were considered as a single group.

**Fig. 1:**
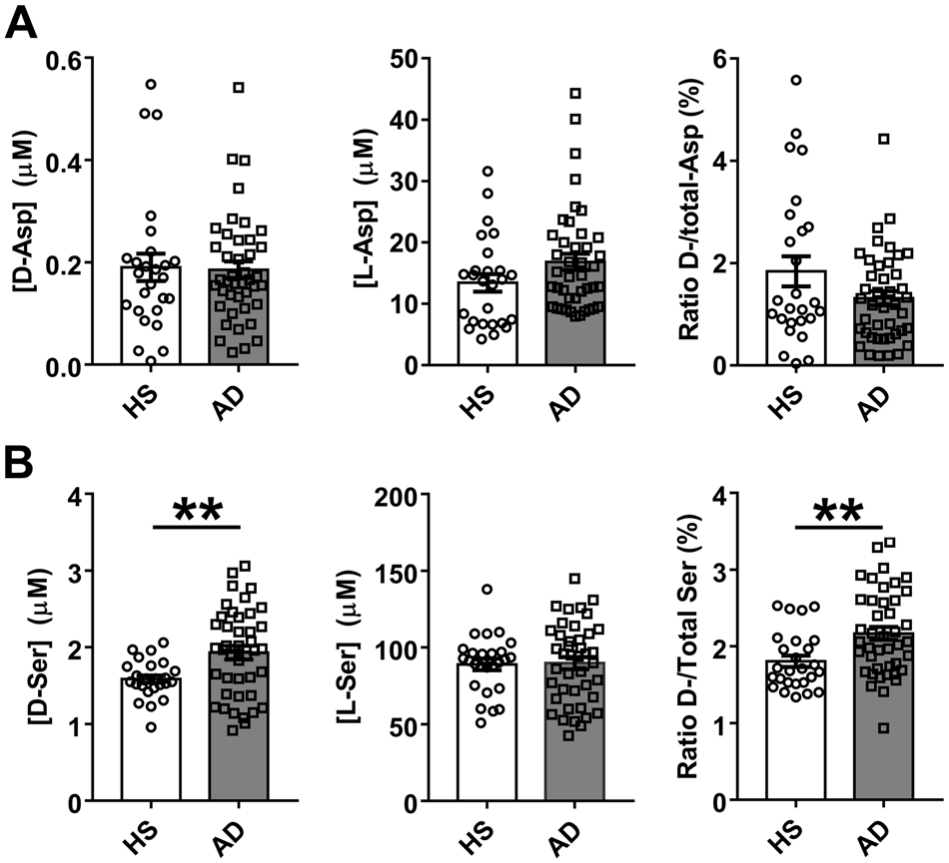
Levels of aspartate and serine enantiomers in human serum. d- and l-Asp (**A**, left and center, respectively) and d- and l-Ser (**B**, left and center, respectively) levels and ratio between d-enantiomer and total (d+l) amino acid content (**A**, right, and **B**, right for Asp and Ser, respectively, expressed as percentage) detected in serum samples of AD patients compared to healthy subjects (HS). Dots and bars represent the single subjects’ values and the standard error of the mean, respectively. **P < 0.01 (Mann–Whitney unpaired test).

**Fig. 2:**
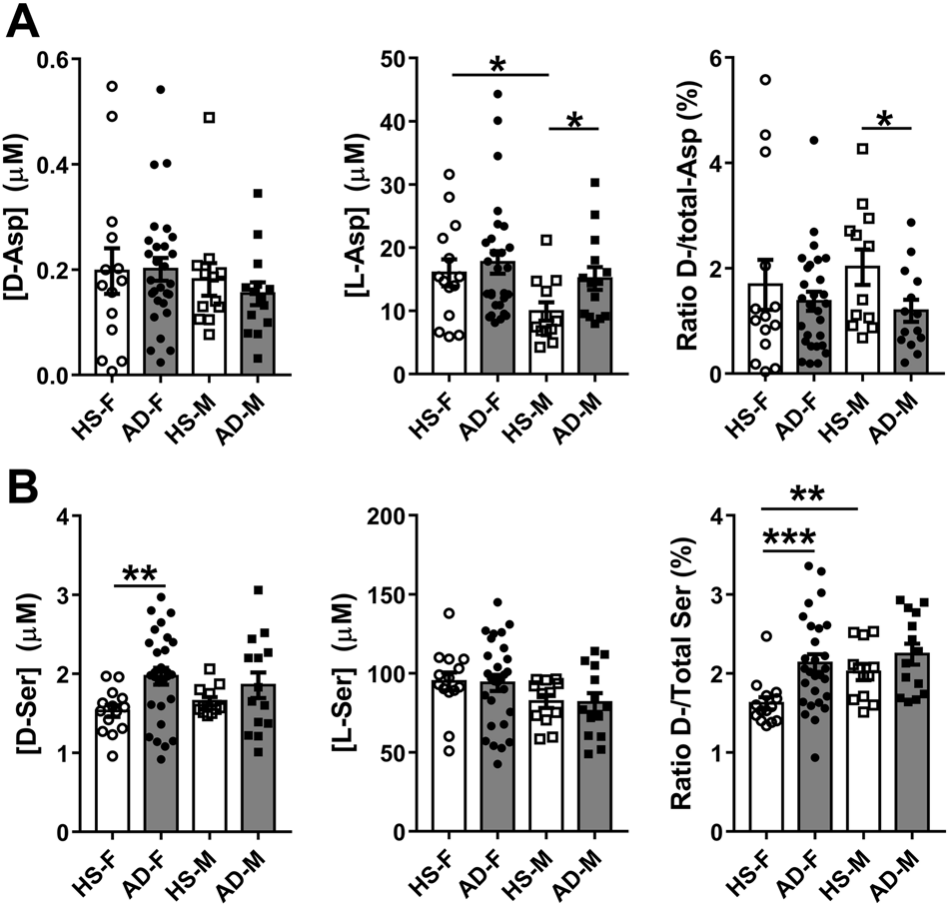
Gender related variations of aspartate and serine enantiomer levels in human serum. d- and l-Asp (**A**, left and center, respectively) and d- and l-Ser (**B**, left and center, respectively) levels and ratio between d-enantiomer and total (d+l) amino acid content (**A** right and **B** right for Asp and Ser, respectively, expressed as percentage) detected in serum samples of AD patients compared to healthy subjects (HS). Dots and bars represent the single subjects’ values and the standard error of the mean, respectively. *P < 0.05; **P < 0.01; ***P < 0.001 (Mann–Whitney unpaired test). F female, M male.

Levels of d-Asp were only slightly decreased in AD patients compared to HS (−2.47%), whereas an increase (+25.3%) was observed for l-Asp: consequently, a decrease (−28.7%) was observed for the d-/total-Asp ratio (Fig. 1 and Supplementary Table 1). These variations resulted not statistically significant. Analyzing the data disaggregated by gender, significant variations between HS and AD patients were found only for the male cohort, in the case of l-Asp and of d-/total-Asp ratio (*P* = 0.0178 and 0.0464, respectively). Minor and no statistically significant differences were found for female cohort. Comparing data of male and female subjects belonging to the same group of subjects (Fig. 2 and Supplementary Table 1), a statistically significant difference was apparent for HS and l-Asp only (*P* = 0.0178), and the relative d-/total-Asp ratio (*P* = 0.0464). The levels of Asp enantiomers were not affected by gender in AD patients.

The d-Ser level showed a statistically significant increase in AD patients with respect to HS (+21.8%, *P* = 0.0060), whereas the l-Ser level was unchanged (Fig. 1B). Similar to d-Ser, a statistically significant increase was observed for the d-/total-Ser ratio (+20.2%, *P* = 0.0025) (Fig. 1 and Supplementary Table 1). A similar pattern was observed comparing cohorts disaggregated by gender: in female cohorts the d-Ser level increased by 28.7%, the d-/total-Ser ratio increased by 31.4%, and the l-Ser level was unchanged. In male cohorts, a lower increase of d-Ser and of d-/total-Ser ratio was observed (+12.3% and +11.2%, respectively) whereas the l-Ser level was unchanged. Comparison between male and female subjects of the same group (Fig. 2 and Supplementary Table 1) revealed a statistical significance only for d-/total-Ser ratio (*P* = 0.0032) of the HS subjects. Similar to d-Asp, gender did not significantly affect the level of Ser enantiomers in AD patients.

### d-Serine levels increase with the progression of the disease in the early stages

The levels of aspartate and serine enantiomers have been compared between homogeneous groups regarding the stage of illness, assessed by the CDR score^39^. AD patients enrolled in this study were at initial stages of AD, corresponding to mild (CDR 1) or moderate (CDR 2) dementia.

**Fig. 3:**
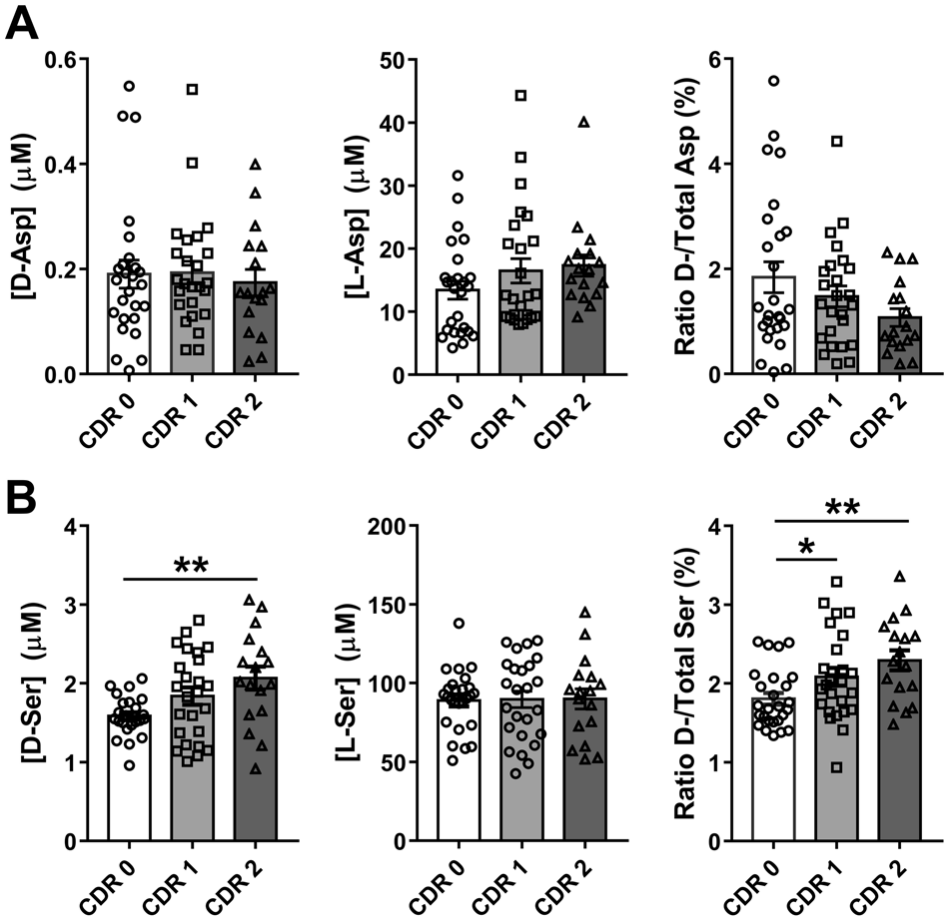
Variations of aspartate and serine enantiomer levels in serum of healthy subjects and AD patients at different stages of the disease. d- and l-Asp (**A** left and center, respectively) and d- and l-Ser (**B** left and center, respectively) levels and ratio between d-enantiomer and total (d+l) amino acid content (**A** right and **B** right for Asp and Ser, respectively, expressed as percentage) detected in serum samples of AD patients compared to healthy subjects (CDR 0) related to the stage of the pathology. Dots and bars represent the single subjects’ values and the standard error of the mean, respectively. *P < 0.05; **P < 0.01 (Mann–Whitney unpaired test). Disease stage was assessed by Clinical Dementia Rating (CDR) score (see Materials and methods): CDR 0: healthy subjects; CDR 1: mild dementia; CDR 2: moderate dementia.

Considering the aspartate levels, comparison of mean levels of analyzed parameters observed for HS, CDR 1, and CDR 2 AD patients did not show any statistical significance (Table 2 and Fig. 3A). However, a modest increase parallel to the gravity of the pathology has been observed for l-Asp together with a parallel decrease in d-/total-Asp ratio (Table 2 and Fig. 3A).

**Table 2:** Statistical analysis of the levels of aspartate and serine enantiomers, d-/total-Asp and d-/total-Ser ratios observed in serum of HS and AD patients.

Amino acid	CDR 1 vs. CDR 0		CDR 2 vs. CDR 1		CDR 2 vs. CDR 0	
	Δ%	*P* value	Δ%	*P* value	Δ%	*P* value
d-Asp	1.58	0.6040	−9.84	0.4733	−8.42	0.9072
l-Asp	23.1	0.2431	4.85	0.2287	29.1	0.0787
Ratio d-/total-Asp	−19.6	0.7017	−27.7	0.2389	−41.8	0.0977
d-Ser	15.9	0.0873	12.5	0.2388	30.2	**0.0015**
l-Ser	0.79	0.7156	0.33	0.8443	1.12	0.9070
Ratio d-/total-Ser	19.4	**0.0336**	10.1	0.2400	26.5	**0.0016**

*CDR 0* healthy subjects, *CDR 1* mild dementia, *CDR 2* moderate dementia. Δ%: variation between compared groups expressed as percentage (Δ% = 100 × ([CDR]_*n*_−[CDR]_*n*−1_)/[CDR]_*n*−1_, where “*n*” is the CDR score, for CDR 1 vs. CDR 0 and CDR 2 vs. CDR 1, and Δ% = 100 × ([CDR]_2_ − [CDR]_0_)/[CDR]_0_ for CDR 2 vs. CDR 0). *P* values obtained with non-parametric Mann–Whitney test are indicated for each comparison. Statistically significant *P* values (*P* < 0.05) are in bold.

Analyses of the results obtained for serine clearly show an increase in the d-Ser levels with the severity of the pathology: statistically significant differences have been found between HS and CDR 2 AD patients' mean values (+30.2%, *P* = 0.0015) (Table 2 and Fig. 3B, left). Since the l-Ser levels remain unchanged (Table 2 and Fig. 3B, center), an increase in the d-/total-Ser ratio was observed: statistically significant variations of this parameter have been found both between HS (CDR 0) and CDR 1 AD patients (+19.4%, *P* = 0.0336) and between HS and CDR 2 AD patients (+26.5%, *P* = 0.0016) (Table 2 and Fig. 3B, right). Analysis of age distribution of HS, CDR 1, and CDR 2 AD patients, carried out using Mann–Whitney test, indicated that a statistically significant difference was observed between HS and CDR 2 AD patients only (*P* = 0.0105), whereas comparison between HS and CDR 1 AD patients and CDR 1 and CDR 2 AD patients was not statistically significant (*P* = 0.5077 and *P* = 0.1012, respectively, see Table 1). These results exclude that the observed increase in the d-Ser serum level was due to differences in age of the cohorts analyzed.

## Discussion

AD is the most common type of dementia in aged people: the pathological changes associated with AD (amyloid deposition and the resulting neuronal death) start decades before the first clinical symptoms appear. Thus, it is crucial to identify and detect parameters indicative of neuropathological changes, eventually those occurring at the synaptic level, in the very precocious stages of the disease. Diagnosis is now based on a neuropsychological evaluation and the assessment of AD biomarkers, such as Aβ oligomers and phosphorylated-tau levels in the CSF. However, CSF examinations are far from standard tests in general practice, due to the invasiveness of the procedure (a lumbar puncture), which also limits this practice for screening and clinical trials. Therefore, there is an urgent need for alternative, accessible peripheral biomarkers, such as serum biomarkers. Accordingly, we monitored the levels of d-Asp and d-Ser, two signaling molecules involved in NMDAR-mediated neurotransmission, in serum from AD patients at early stages of illness (CDR 1 or 2) and age-matched controls (CDR 0). We employed a standardized, well-validated analytical procedure based on pre-column derivatization, HPLC separation, and quantification of the d-enantiomers by enzymatic degradation.

d-Asp and l-Asp serum content did not show any statistically significant variation between AD patients and HS, despite a reasonable increase in l-Asp levels (≈25%) was observed in AD patients. By analyzing data disaggregated by gender, statistically significant differences were observed for l-Asp only: in the HS male cohort the l-Asp levels were significantly lower compared to the corresponding AD cohort and to female ones (independently from the diagnosis). As a consequence, in the AD male cohort the d-/total-Asp ratio appeared significantly decreased compared to controls. On the other hand, statistically significantly elevated d-Ser serum levels (≈20%) were detected in AD patients with respect of HS. Data disaggregated by gender indicate a higher increase in female cohorts (≈30%) compared to male (≈10%) cohorts, despite in this latter case the difference between AD patients and HS was not statistically significant. No significant change depending on diagnosis and/or gender is observed in l-Ser serum levels, while the d-/total-Ser ratio is higher in male compared to female HS cohorts. Overall, our results point to a slight gender effect on the serum levels of both d- and l-Asp and d- and l-Ser, thus confirming previous results obtained in ventricular CSF^25^ and in post mortem tissues and CSF^30^. On the other hand, a significant positive correlation between serum d-serine levels and age has been observed in AD patients and not in HS, despite a negative association previously reported^39^.

Separating subjects into experimental groups stratified by the CDR score showed an increasing trend for l-Asp content from cognitively HS (CDR 0) to patients with a CDR score of 2, which resulted into a specular trend in the d-/total-Asp ratio values. Lack of statistical significance for d-Asp might be ascribed to its very low concentration (0.15–0.2 µM), close to the limit of detection. Conversely, serum d-Ser levels paralleled the progression of the pathology: the lowest levels were detected in HS and increasing levels were observed in AD patients, being significantly higher in individuals with a CDR of 2. This positive correlation is even more evident for d-/total-Ser ratio. In this latter case, a statistically significant increase is also evident in patients with a CDR score of 1, suggesting this ratio as a more sensitive parameter of the pathology and its progression than absolute d-Ser levels.

This latter result is in line with recent observations reporting increased d-serine levels in some areas of AD brains involved in the disease progression^30^, likely due to upregulation of SR expression in reactive neurotoxic astrocytes^27^ and the consequent increased d-Ser release. Worthy of note, converging lines of evidence indicate reduced levels of NMDAR in the same brain regions in AD patients^40,41^. It has been suggested that elevated levels of the co-agonist d-Ser might underlie a mechanism by which Aβ oligomers trigger synapse dysfunction and the resulting memory impairment^30^: Aβ-induced d-Ser increase might represent an initial adaptive response to maintain proper neurotransmission in the early stages of the disease^42^, but, since NMDARs appear overactivated in AD, they can also contribute to the excitotoxic scenario in later stages, worsening the neuropathological outcomes. d-Ser inhibits apoptosis at early phases and stimulates necrosis at later phases^43^. The development of amyloid plaques typically precedes clinically significant cognitive symptoms by at least 10–15 years: a time course comparison between plaques formation and d-Ser levels could allow to elucidate the events bringing to the dementia. Future studies should also evaluate the correlation between d-Ser levels and peripheral biomarkers of AD, i.e. blood levels of Aβ42, phosphorylated tau, etc.

As a next step in the field, analyses on subjects showing amnestic mild cognitive impairment (MCI, CDR = 0.5) and with a significantly increased probability to develop AD will allow to validate the use of serum serine levels as a valuable biomarker of the disease onset. To be enrolled in this study, amnestic MCI patients need to undergo imaging analysis (e.g., PET) to verify the presence of a neurodegenerative process.

## Conclusions

Considerations related to the effect of Aβ on the d-Ser synthesis/level, and thus on NMDAR functioning, in AD pathophysiology and our results concerning serine levels (determined by a procedure based on enzymatic selective degradation of the d-enantiomer) strengthen the idea of serum d-Ser levels and/or d-/total-Ser ratio as a valuable (and simple to assay) biological marker for AD to evaluate the disease progression as well as the precocious stages of the illness. Accordingly, we suggest the combined use of blood-based biomarkers currently under development^44^ and serine enantiomers concentration ratio as a novel and relevant strategy to increase sensitivity and specificity in the diagnosis of AD.

## Supplementary information

Supplemental material
