# Supplementary material for: Serum d-serine levels are altered in early phases of Alzheimer’s disease: towards a precocious biomarker
Source: Transl Psychiatry. 2021 Jan 26;11:77. doi: 10.1038/s41398-021-01202-3 (PMC7838302; doi:10.1038/s41398-021-01202-3)
Supplement: Supplementary file 1 — Supplemental material [file 41398_2021_1202_MOESM1_ESM.docx]

**Supplementary Materials**

**Serum D-serine levels are altered in early phases of Alzheimer’s disease: towards a precocious biomarker**

Luciano Piubelli^a*^, Loredano Pollegioni^a^, Valentina Rabattoni^a^, Marco Mauri^a,b^, Lucia Princiotta-Cariddi^c,d^, Maurizio Versino^b,d^, Silvia Sacchi^a^

^a^Department of Biotechnology and Life Sciences, University of Insubria, Varese, Italy

^b^Neurology Unit, Ospedale di Circolo and Fondazione Macchi, ASST Settelaghi, Varese, Italy

^c^Center of Research in Medical Pharmacology, University of Insubria, Varese, Italy

^d^Department of Medicine and Surgery, University of Insubria, Varese, Italy





**Supplementary Figure 1:** Age-related distribution of D- (A) and L-Asp (B) and D- (D) and L-Ser (E) levels and of ratio between D-enantiomer and total (D+L) amino acid content (C and F for Asp and Ser, respectively; expressed as percentage) detected in serum samples of AD patients (open circles) and of healthy subjects (closed circles). Dots represent the single subjects’ values.

**Supplementary Table 1.** Serum levels of D,L-aspartate, D,L-serine, D-/total-Asp and D-/total-Ser ratios and comparison between HS and AD patients.

| **Subjects** | | **Nume-rosity** | **D-Asp (µM)** | |  | **L-Asp (µM)** | |  | **Ratio D-/total**-**Asp (%)** | |
| --- | --- | --- | --- | --- | --- | --- | --- | --- | --- | --- |
|  |  |  | **Mean ± SD (SEM) [RE%]** | **Δ% (P-value)** |  | **Mean ± SD (SEM) [RE%]** | **Δ% (P-value)** |  | **Mean ± SD (SEM) [RE%]** | **Δ% (P-value)** |
| All | HS | 26 | 0.190 ± 0.137 (0.0268) [71.9] | -2.47 (0.7756) |  | 13.4 ± 7.3 (1.43) [54.3] | 25.3 (0.0928) |  | 1.84 ± 1.51 (0.295) [81.8] | -28.7 (0.2702) |
|  | AD | 42 | 0.185 ± 0.106 (0.0163) [56.9] |  |  | 16.8 ± 8.6 (1.32) [51.0] |  |  | 1.30 ± 0.89 (0.135) [68.0] |  |
| Female | HS | 14 | 0.197 ± 0.161 (0.0431) [81.8] | 1.72 (0.6974) |  | 16.0 ± 8.0 (2.13) [49.7] | 10.4 (0.8176) |  | 1.69 ± 1.78 (0.475) [105] | -18.6 (0.6977) |
|  | AD | 28 | 0.201 ± 0.114 (0.0216) [56.8] |  |  | 17.7 ± 9.4 (1.77) [53.0] |  |  | 1.37 ± 0.95 (0.180) [69.3] |  |
| Male | HS | 12 | 0.181 ± 0.107 (0.0310) [59.2] | -14.9 (0.5952) |  | 9.90 ± 5.02 (1.449) [50.7] | 52.8 (0.0178) |  | 2.02 ± 1.16 (0.336) [57.6] | -41.0 (0.0464) |
|  | AD | 14 | 0.154 ± 0.081 (0.0216) [52.3] |  |  | 15.1 ± 6.7 (1.79) [44.3] |  |  | 1.19 ± 0.78 (0.209) [65.7] |  |

**Supplementary Table 1. (continued)**

| **Subjects** | | **Nume-rosity** | **D-Ser (µM)** | |  | **L-Ser (µM)** | |  | **Ratio D-/total**-**Ser (%)** | |
| --- | --- | --- | --- | --- | --- | --- | --- | --- | --- | --- |
|  |  |  | **Mean ± SD (SEM) [RE%]** | **Δ% (P-value)** |  | **Mean ± SD (SEM) (RE%)** | **Δ% (P-value)** |  | **Mean ± SD (SEM) (RE%)** | **Δ% (P-value)** |
| All | HS | 26 | 1.59 ± 0.24 (0.048) [15.3] | 21.8 (0.0060) |  | 89.0 ± 19.1 (3.74) [21.4] | 0.88 (0.7565) |  | 1.81 ± 0.37 (0.073) [20.7] | 20.2 (0.0025) |
|  | AD | 42 | 1.93 ± 0.57 (0.088) [29.6] |  |  | 89.8 ± 26.0 (4.01) [28.9] |  |  | 2.17 ± 0.55 (0.085) [25.5] |  |
| Female | HS | 14 | 1.53 ± 0.28 (0.075) [18.3] | 28.7 (0.0070) |  | 94.9 ± 21.2 (5.66) [22.3] | -1.0 (0.9475) |  | 1.62 ± 0.29 (0.077) [17.8] | 31.4 (0.0007) |
|  | AD | 28 | 1.97 ± 0.56 (0.106) [28.6] |  |  | 93.9 ± 27.0 (5.11) [28.8] |  |  | 2.13 ± 0.59 (0.111) [27.5] |  |
| Male | HS | 12 | 1.65 ± 0.18 (0.052) [10.9] | 12.3 (0.5019) |  | 82.2 ± 14.1 (4.08) [17.2] | -0.79 (0.9798) |  | 2.02 ± 0.36 (0.103) [17.7] | 11.2 (0.2574) |
|  | AD | 14 | 1.86 ± 0.60 (0.161) [32.5] |  |  | 81.5 ± 22.4 (5.98) [27.4] |  |  | 2.25 ± 0.49 (0.132) [22.0] |  |

**Supplementary Table 1. (continued)**

HS: healthy subjects. D-/total-Asp and D-/total-Ser ratio are expressed as percentage. Data are Mean ± Standard Deviation (SD). The Standard Errors of the Mean (SEM) and the Relative Errors, expressed as percentage (RE% = 100x(SD/Mean)), are reported in parentheses. Δ%: variation between mean values of AD and HS expressed as percentage (Δ% = (([AD]_Mean_ – [HS]_Mean_)/[HS]_Mean_)x100). P-values obtained with non-parametric Mann-Whitney test are indicated for each comparison. Statistically significant P-values (P < 0.05) are underlined.

**Supplementary Table 2.** Serum levels of D,L-aspartate, D,L-serine, D-/total-Asp and D-/total-Ser ratios observed in HS and AD patients.

| **Group** | **CDR** | **Nume-rosity** | **Mean ± SD (SEM) (µM); [RE%]** | | | | | |
| --- | --- | --- | --- | --- | --- | --- | --- | --- |
|  |  |  | **D-Asp** | **L-Asp** | **Ratio D**-**/ total Asp** | **D-Ser** | **L-Ser** | **Ratio D**-**/ total**-**Ser** |
| Healthy subjects | 0 | 26 | 0.190 ± 0.137 (0.0267) [71.9] | 13.4 ± 7.3 (1.43) [54.3] | 1.84 ± 1.51 (0.295) [81.8] | 1.59 ± 0.24 (0.048) [15.3] | 89.0 ± 19.1 (3.74) [21.4] | 1.81 ± 0.37 (0.073) [20.7] |
| Mild dementia (CDR 1) | 1 | 25 | 0.193 ± 0.108 (0.0216) [56.0] | 16.5 ± 9.6 (1.93) [58.5] | 1.48 ± 0.98 (0.197) [66.7] | 1.84 ± 0.55 (0.109) (29.7] | 89.7 ± 26.2 (5.24) (29.2] | 2.08 ± 0.57 (0.114) [27.2] |
| Moderate dementia (CDR 2) | 2 | 17 | 0.174 ± 0.104 (0.0251) [59.6] | 17.3 ± 7.0 (1.69) [40.2] | 1.07 ± 0.70 (0.169) [65.2] | 2.07 ± 0.60 (0.145) [29.0] | 90.0 ± 26.5 (6.42) [29.4] | 2.29 ± 0.52 (0.127) [22.8] |

**Supplementary Table 2 (continued).**

HS: healthy subjects. D-/total-Asp and D-/total-Ser ratio are expressed as percentage. Data are Mean ± Standard Deviation (SD). The Standard Errors of the Mean (SEM) and the Relative Errors, expressed as percentage (RE% = 100x(SD/Mean)), are reported in parentheses.
